# Are there any preventable risk factors for women who had surgery for Pelvic Organ Prolapse and stress Urinary Incontinence?

**DOI:** 10.12669/pjms.344.14944

**Published:** 2018

**Authors:** Sefa Kurt, Mehmet Tunc Canda, Mehmet Bal, Abdullah Tasyurt

**Affiliations:** 1 Dr. Sefa Kurt, Department of Obstetrics and Gynecology, Dokuz Eylul University, Izmir, Turkey; 2 Dr. Mehmet Tunc Canda, Obstetrics and Gynecology Unit, Kent Hospital, Izmir, Turkey; 3 Dr. Mehmet Bal, Obstetrics and Gynecology Unit, Tepecik Teaching Hospital, Izmir, Turkey; 4 Dr. Abdullah Tasyurt, Obstetrics and Gynecology Unit, Tepecik Teaching Hospital, Izmir, Turkey

**Keywords:** Incontinence surgery, Pelvic organ prolapse, Prolapse surgery, Risk factors, Stress urinary incontinence

## Abstract

**Objective:**

The present study evaluates the preventable risk factors in symptomatic women with previous surgery for pelvic organ prolapse (POP) and/or stress urinary incontinence (SUI).

**Methods:**

Four hundred and one women previously operated were divided into two groups as; women operated for POP (325 cases) and women operated for SUI (76 cases). The control group consisted of 233 age and body mass index (BMI)-matched subject operated for benign gynecologic reasons and exhibited no evidence of POP or SUI. These groups were compared in terms of age, BMI, gravida, parity, mode of delivery, smoking status, menopause status and chronic diseases.

**Results:**

Grand_multiparity (parity ≥5) increased the risk of POP/SUI surgery and POP surgery 2.71 and 2.94 times, respectively (p=0.0003 and p=0.0001, respectively). Vaginal birth increased the risk of POP/SUI surgery 2.33 times (p=0.03).

**Conclusion:**

Grand_multiparity increased the risk of POP/SUI surgery and POP surgery while vaginal birth increased the risk of POP/SUI surgery. Among them, particularly, grand_multiparity seem to be the only preventable risk factors.

## INTRODUCTION

Pelvic floor disorders are a bothersome health problem of the adult female population, and the lifelong chance of going through an operation for pelvic organ prolapse (POP) and/or urinary incontinence (UI) estimated to be 11.1%, 12.1% and 19% in the United States (US)^1^, in the United Kingdom 2 and Australia^3^, respectively. The reoperation risks for POP and/or UI vary from 19%- 29%.^1^,^2^ A study from Australia reported prevalence rates of 8.8% for POP and 20.8% for stress urinary incontinence (SUI) in the female population.^4^. Another study from Europe reported an 8.3% prevalence of symptomatic POP and an 8.9% prevalence of SUI.^5^ A study from Turkey reported a prevalence of 7.9% for SUI.^6^ In the US, POP and incontinence surgeries cost more than $1 billion per year.^1^ As the number of aging women increases, the expected treatment costs will also increase in the coming decades.

Previously described risk factors for developing POP and incontinence included advanced age, white race, obesity, vaginal delivery, increased intra-abdominal pressure, smoking, connective tissue disorders, previous hysterectomy, having a mother with POP and POP symptoms during pregnancy.^7^,^8^ Several studies have utilized questionnaires to determine the risk factors for POP in the general population and included both asymptomatic and symptomatic women; however, only a small portion of the participants in these studies underwent POP and/or UI surgery or a gynecologic exam to define the exact grades of POP.^4^,^8^-^10^ Few studies have investigated the risk factors that predispose women who had undergone surgery for POP and incontinence surgery.^1^,^2^,^11^

The present study define the risk factors of pelvic floor disorders particularly, in symptomatic women with previous surgery for POP and SUI based on patient records through comparison with the records of women with previous gynecologic operations for benign reasons and exhibited no evidence of POP and/or SUI.

## METHODS

The records of patients with previous gynecologic operation between 2011 and 2012 were investigated, and the records of women who had undergone operations for POP and SUI retrospectively were analyzed. An age and body-mass index (BMI)-matched control group was chosen from the patient pool of women who had undergone operations for benign gynecologic reasons and exhibited no evidence of POP and SUI both by examination and by questionnaires. All participants answered questions related to the short forms of Pelvic Floor Distress Inventory (PFDI-20) and Pelvic Floor Impact Questionnaire (PFIQ-7).^12^ The study was approved by the Research Ethics Committee of the hospital.

The study group was divided into the following two groups: Group-1 was composed of women with POP; Group-2 was composed of women with stress UI (SUI). The study and control groups were assessed in terms of age, BMI, gravidity, parity, mode of delivery (vaginal birth, cesarean section and cesarean section following a vaginal birth), smoking status, menopause status and chronic diseases (e.g., hypertension, diabetes, neurologic diseases, and chronic lung disease, etc). These data was collected according to previously published recommendations.^13^ In this study multiparity is defined as parity between two to four and grand_multiparity is defined as parity ≥5.

Pelvic organ prolapse staging performed according to the Baden-Walker halfway system.^14^ Pelvic organ prolapse includes anterior vaginal prolapse (cystocele), apical or uterine prolapse and posterior vaginal prolapse (rectocele). Women diagnosed and operated for POP were included in the study. Stress urinary incontinence was defined according to the standard definitions developed by the International Continence Society.^15^ The patient’s statement of involuntary urine loss during physical activity or coughing was defined as SUI. Urge and mixed incontinence cases were excluded from the study. To minimize the interobserver variability between examiners, all cases opted for an operation examined by the same researcher prior to the operation and the final staging recorded.

### Statistical Analysis

According to a previous study^16^, a minimum sample size of 233 is required to achieve an 80% power to detect a 2-fold difference in parity between groups with a significance level of 0.05.

The control group created on 1:1 matching for age and BMI in a random format with using SAS 9.1 (SAS Institute Inc., Cary, NC, USA). The data presented as numbers (percentages) or means ± standard deviations as appropriate. As descriptive statistics, the means ± SDs, minimum and maximum values and 95 % confidence intervals (CIs) of the mean determined. Numerical data analyzed using one-way Anovas. The Kruskal- Wallis test used to compare variable across groups. Chi-square tests used to analyze qualitative variances. Logistic regression used for univariate and multivariate analysis.

Odds ratios (OR) and 95% CIs presented for the factors associated with previous POP and/or SUI surgery. *P* < 0.05 was considered as statistically significant. The data analyzed with the SPSS (release 16.0).

## RESULTS

The study group consisted of 401 subjects with adequate data and included the POP group (325 subjects) and the SUI group (76 subjects). The number of women who experienced POP and SUI symptoms were 40. The control group consisted of 233 age and BMI-matched women who had gynecologic operations for benign reasons. A total of 634 subjects were included in the study. The women in the study group had POP grade two or higher, while the control group had POP grade 0 or one according to the Baden-Walker halfway system. There were no significant differences between the study and control groups in terms of age, BMI, mean gravidity or mean parity as summarized in Table-I. Only the POP group was significantly older than the SUI group in terms of mean age (p<0.001). The general characteristics of each group are summarized in Table-II. Multiparity, grand-multiparity, vaginal birth, hypertension and chronic diseases are significantly more common in the study group than the control group. Risk factors that were associated with previous POP and/or SUI surgery are summarized in Table-III. Grand_multiparity and vaginal birth are the primary risk factors associated with POP and/or SUI surgery.

**Table-I:** Comparison of the baseline characteristics of the groups.

	Study (POP/SUI) group (n= 401)	POP group (n=325)	SUI group (n=76)	Control group (n=233)	P-value; 95% CI
Age (years)	54.7±11.6 (35-83)	56.2±11.5 (35-83)	48.6±11 (37-80)	54.3±7.3 (35-68)	p<0.001; 3.74-11^a^
BMI	27.6±3.5 (18-40)	27.92±3.8 (18-40)	26.12±3.9 (19-37)	27.72±2.92 (23-38)	p=0.033; 0.12-3.48^a^
Gravidity	4,9±2.6 (0–15)	4.94±2.6 (0-14)	4.59±2.6 (1-15)	4.5±2.1 (2-12)	N.S
Parity	3.84±2.1 (0-12)	3.89±2.1 (0-12)	3.62±1.9 (0-11)	3.32±1.7 (2-9)	N.S

^a,b^between POP and SUI groups.

**Table-II:** Comparison of general characteristics between groups.

	Study group (POP/SUI) (n=401)	POP group (n=325)	SUI group (n=76)	Control Group (n=233)	P-value^a^
Nulliparity	5 (1.2%)	4 (1.3%)	1 (1.8%)	2 (0.8%)	NS
Primiparity	11 (2.7%)	10 (3.3%)	1 (1.8%)	3 (1.2%)	NS
Multiparity	279 (69.5%)	222 (68.3%)	57 (75%)	200 (85.8%)	0.0001
Grand_multiparity	106 (26.4%)	90 (27.6%)	16 (21%)	28 (12%)	0.0003
Vaginal birth	378 (94.2%)	310 (95.3%)	68 (89.4%)	209 (89.6%)	0.03
Caesarean section	4 (0.9%)	3 (0.9%)	1 (1.3%)	8 (3.4 %)	NS
Vaginal birth + caesarean section^b^	19 (4.73%)	14 (4.3%)	5 (6.5%)	16 (6.8%)	NS
Menopausal status	277 (69%)	241 (74.1%)	36 (47.3%)	96 (41%)	<0.0001
Hypertension	127 (31.6%)	108 (33.2%)	19 (25%)	66 (28.3 %)	<0.0001
Diabetes mellitus	43 (10.7%)	36 (11%)	7 (9.2%)	21 (9%)	NS
Pulmonary disease	17 (4.2%)	12 (3.6%)	5 (7%)	8 (3.4%)	NS
Neurological disease	12 (2.9%) 11	11 (3.3%)	1 (1.8%)	3 (1.2 %)	NS
Chronic diseases^c^	175 (43.6%)	157 (48.3%)	28 (36.8%)	77 (33%)	0.01
Smoking	35 (8.7%)	26 (8%)	9 (11.8%)	19 (8.1%)	NS

NS: not significant,

a p value between study and control groups,

b women who had caesarean section following a previous vaginal birth were given,

c chronic diseases included hypertension, diabetes mellitus, pulmonary disease and neurologic disease.

**Table-III:** Risk factors associated with previous POP and/or SUI surgery.

	Univariate analysis OR (95% CI)	Multivariate analysis OR (95% CI)
Age>50	2.63 (1.86-3.72)^a^	
BMI>25	0.57 (0.40-0.88)^a^	
Multiparity	0.38 (0.24-0.59)^a^ 0.36 (0.22-0.57)^b^	
Grand_multiparity	2.63 (1.64- 4.25)^a^ 2.83 (2.73-4.64)^b^	2.71 (1.61-4.45)^a^ 2.94 (2.5-5.24)^b^
Vaginal births including instrumental deliveries	2.58 (1.23-5.49)^b^	2.33 (1.1-4.36)^a^
Caesarean section	0.28 (0.07-1.05)^a^	
Menopause	3.19 (2.25-4.53)^a^ 3.85 (2.62-5.67)^b^	2.62 (1.43-4.57)^a^ 3.13 (2.14-4.91)^b^
Hypertension	2.54 (1.65-3.92)^a^ 2.63 (1.68- 4.13)^b^	
Chronic diseases^c^	1.57 (1.10-2.23)^a^ 1.65 (1.14-2.39)^b^	

a between study and control group,

b between POP and control group

c chronic diseases included hypertension, diabetes mellitus, pulmonary disease and neurologic disease

## DISCUSSION

The present study demonstrated that grand_multiparity and vaginal birth were important risk factors for future POP and SUI surgery. The importance of these attributed risk factors comes from that they were the results of investigating a strict group of patients with previous POP and/or SUI surgery rather than investigating a general population only with questionnaires.

Parity always investigated as a risk factor for POP and/or SUI. A previous study reported that the risk of pelvic floor dysfunction is not further increased by parity>3.^17^ MacArthur et al.^18^ reported that parity≥4 increases the risk of UI, and Abdel-Fattah et al.^2^ reported that parity between 2 to 4 is an independent risk factor for POP/UI surgery. Additionally, the present study found that vaginal birth was another important risk factor for future POP and SUI surgery. This result agrees with those of some previous studies.^2^,^4^,^16^ Some studies reported that abdominal deliveries are protective against pelvic floor dysfunction, but we did not reach this conclusion^2^,^5^,^19^

de Boer et al.^11^ reported that women who have previously undergone POP/incontinence surgeries are typically postmenopausal. Additionally, another study stated that menopause predisposes women to prolapse of their pelvic organs.^20^ Our results did not find an association between menopausal status and POP/SUI surgery.

The findings of the present study demonstrated that POP and SUI surgeries performed at approximately at the age of 54. Particularly POP surgery performed at approximately the age of 56 and SUI surgery performed at approximately the age of 48. Two of the previous questionnaire-based studies reported no associations of increasing age with POP, and the only association reported in these studies was that between increased age and UI.^8^,^21^ In another study increased age was reported to be associated with UI and POP.^4^ Additionally, a study that compared women with POP symptoms evaluated for POP stages to asymptomatic controls reported that the age of the POP group was higher.^22^ Furthermore, a study that reported on the characteristics of women who had previously undergone POP/incontinence surgeries found that these women were older.^11^ The multivariate analysis did not reveal an association of age>50 with POP and SUI surgeries in the present study.

Some of the previous studies have reported that increased BMI is a risk factor for undergoing POP and/or incontinence surgery.^1^,^2^,^11^ However, some questionnaire-based studies did not reach similar conclusions.^8^,^21^ Prior studies evaluating age and BMI as risk factors for POP and SUI have been inconsistent. In addition, the multivariate analysis did not reveal an association of BMI>25 with POP and SUI surgeries in the present study.

The women who had undergone POP and SUI surgeries were more hypertensive and/or more likely to have accompanying chronic diseases, but these conclusions were not found to be significant in the multivariate analysis. Previous studies have not reported on these relationships, and only two previous studies have reported on the association between chronic lung disease and POP/UI surgery.^1^,^16^

According to the results of the present study, we suggest that the clinicians may warn their patients who reached to four births about the strong evidence of developing POP or SUI in the future if they wish to give five or more births and particularly by vaginal route.

Many previous studies have investigated the general characteristics and risk factors for POP and/or SUI according to questionnaire-based prevalence studies that including information about prior surgeries and some of these studies lacked information about POP staging.^4^,^8^-^10^ This lack of information may have led to the exaggeration of some symptoms, over or underestimation of the risk factors and misleading conclusions. The strength of the present study is that we investigated the general characteristics and possible associated risk factors of women who had undergone operations of POP and/or SUI. The evaluation of such targeted group significantly increased the credibility of this study.

### Limitations

Our study had several limitations. Firstly, our study is a retrospective study. Secondly, the number of the cases in the whole study group (401), POP group (325) and the control group (233) were adequate in terms of power calculation, but SUI (76) group was limited. Thirdly, even the study and the control groups were age and BMI-matched, they were not homogenously distributed in terms of gravidity, parity, mode of delivery, menopausal status, chronic diseases and smoking status. However, there was no statistical difference in terms of age, BMI, gravidity, parity, menopausal status and smoking status between the study and control groups (Table I and II). On the other hand, we assessed a local population of Caucasian women so it may be hard to compare our results to other ethnicities. In addition, we did not study the re-operation rates and the underlying causes. From the methodological point of view, we used the Baden-Walker halfway system for staging of POP, however a more current and reliable staging system known as pelvic organ prolapse-quantification system may be preferred.

## CONCLUSION

The results of the current study revealed that grand_multiparity and vaginal delivery are important risk factors for POP and/or SUI surgeries. Among these risk factors, grand_multiparity appears to be the only preventable risk factor.

### Author`s Contribution

**SK** conceived the idea, data supervision, writing and critical review of the manuscript.

**MTC** Designed the study, data analysis and interpretation, writing of the manuscript.

**MB** did data collection, processing and literature review.

**AT was** involved in conception of the study, final approval of the manuscript.
